# Synthesis, characterization, and application of MnFe_2_O_4_/FeVO_4_/modified zeolite nanocomposite as an effective photocatalyst for methylene blue degradation and benzothiophene desulfurization

**DOI:** 10.1016/j.heliyon.2024.e41294

**Published:** 2024-12-16

**Authors:** Shima H. Khabbaz, Ahmad Bagheri, Mehdi Mousavi-Kamazani

**Affiliations:** a Department of Chemistry, Semnan University, P.O. Box 35131-19111, Semnan, Iran; b Department of Nanotechnology, Faculty of New Sciences and Technologies, Semnan University, Semnan, Iran

**Keywords:** MnFe_2_O_4_/FeVO_4_/Modified zeolite, Nanocomposite, Photocatalyst, Methylene blue, Desulfurization

## Abstract

This study details the synthesis of a novel ternary nanocomposite composed of MnFe_2_O_4_, FeVO_4_, and modified zeolite, achieved through a two-step process. The initial step involved the hydrothermal synthesis of the MnFe_2_O_4_/FeVO_4_ composite, followed by its application onto modified zeolite using ultrasonic waves. The synthesized nanocomposite was thoroughly characterized using a range of analytical techniques. X-ray diffraction (XRD) analysis indeed confirmed the successful synthesis of MnFe_2_O_4_/FeVO_4_ composite without impurities and other by-products. This purity was significantly facilitated by the inclusion of hydrazine during the synthesis process. Field emission scanning electron microscopy (FESEM) provided insights into the morphology, indicating that the use of hydrazine resulted in smaller particle sizes and reduced agglomeration. Energy dispersive spectroscopy (EDS) mapping demonstrated a uniform distribution of the particles on the modified zeolite, while transmission electron microscopy (TEM) images revealed spherical particle shapes and the layered structure of the zeolite. Diffusion reflectance spectroscopy (DRS) analysis indicated bandgap values of 1.38 eV for MnFe_2_O_4_**/**FeVO_4_, 1.48 eV for the 1:1 ratio of MnFe_2_O_4_**/**FeVO_4_/modified zeolite, and 1.44 eV for the 2:1 ratio. In terms of pollutant degradation, the study evaluated the removal efficiency of methylene blue (MB) across various samples, with the following removal percentages: MnO_2_/MnFe_2_O_4_ (66 %), FeO_2_/Fe_0.5_V_3.5_O_8_ (60 %), MnFe_2_O_4_**/**FeVO_4_ (67 %), amorphous (58 %), MnFeO_3_/Fe_2_VO_4_/Mn_2_V_2_O_7_ (64 %), MnFe_2_O_4_**/**FeVO_4_/modified zeolite (1:1) (84 %), and MnFe_2_O_4_**/**FeVO_4_/modified zeolite (2:1) (93 %). For sulfur removal, the efficiencies were recorded as 67 %, 28 %, 68 %, 45 %, 22 %, 83 %, and 100 % for the same samples, respectively. This comprehensive characterization and evaluation of pollutant removal efficiencies highlight the potential applications of the synthesized nanocomposite in environmental remediation.

## Highlights

• MnFe_2_O_4_/FeVO_4_/modified zeolite photocatalyst was synthesized for the first time.
• MnFe_2_O_4_/FeVO_4_ particles were synthesized by hydrothermal method and spread on modified zeolite using sonochemical method.
• MnFe_2_O_4_/FeVO_4_/modified zeolite photocatalyst can degrade methylene blue up to 93 % and remove sulfur up to 100 % in less than 30 min.
• The MnFe_2_O_4_/FeVO_4_/modified zeolite photocatalytic process follows Sips and Khan adsorption kinetics.
• The MnFe_2_O_4_/FeVO_4_/modified zeolite photocatalytic process showed good reusability after 3 cycles.

## Graphical abstract

**Figure:**
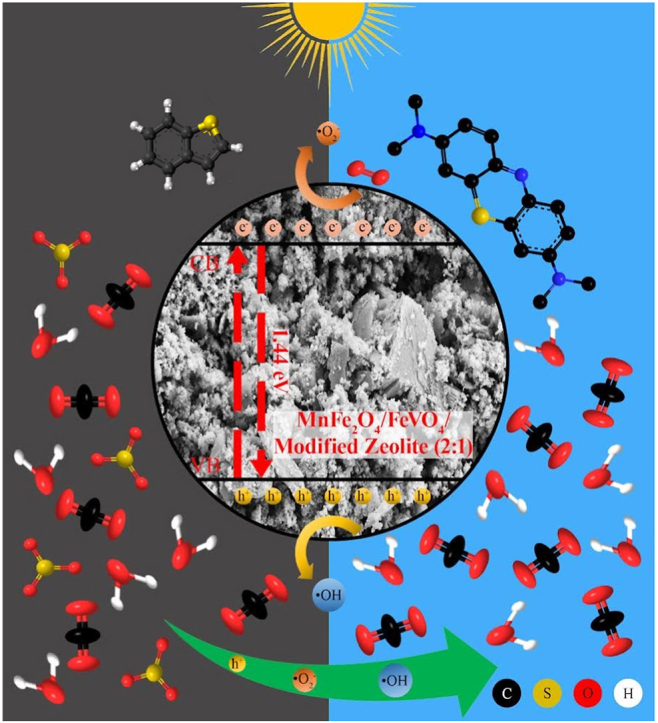

## 1 Introduction

With the increase in population and advancements in technology, environmental pollution has become one of the most significant global challenges [1,2]. Today, pollution is one of the most pressing issues we face [1,3]. It is typically categorized into three main types: air, water, and land pollution. Examples of pollutants include organic dyes, sulfur, and others [[3], [4], [5]]. These pollutants pose a range of threats to both the environment and human health [5,6]. Some of the problems they cause include equipment corrosion [7], catalyst deactivation [7,8], respiratory issues [9], skin diseases [4,10], and the destruction of ecosystems, habitats, and biodiversity [[11], [12], [13]]. To mitigate environmental pollution and safeguard human health, researchers have explored various methods for pollutant removal [14]. For instance, the removal of organic dyes is generally classified into three main categories: physical methods like membrane processes [1,6,14], chemical methods such as advanced oxidation and photocatalysis [15,16], and biological methods including aerobic and anaerobic filtration [17,18]. Each of these methods has its own set of advantages and limitations [14], and the choice of method must be justified economically and in terms of safety [19]. Another critical pollutant is sulfur in hydrocarbons [20]. To reduce sulfur and improve gasoline quality [21], several desulfurization techniques are employed, such as adsorption desulfurization (ADS) [22], extraction desulfurization (EDS) [23], hydrodesulfurization (HDS) [24,25], biological desulfurization (BDS) [26], oxidation desulfurization (ODS) [27], and photocatalytic oxidative desulfurization (PODS) [28,29]. As noted, numerous methods exist for pollutant removal; however, it is essential to select one that balances economic feasibility with safety for both human and animal health. For example, HDS requires high temperatures and pressures, while EDS and ADS incur significant solvent and adsorbent costs. Consequently, researchers recommend photocatalytic oxidative desulfurization (PODS) [7,11,24,28]. Recently, PODS has gained global attention for its cost-effectiveness and eco-friendly nature. Researchers have achieved efficient degradation by using photocatalysts to generate highly reactive radicals that break C-S-C bonds in sulfur compounds [30]. These free radicals possess sufficient oxidation and reduction capabilities to convert sulfur-containing pollutants into less harmful or non-toxic substances [31]. The structure of nanomaterials significantly influences their properties, especially in photocatalytic systems [32]. Under light irradiation, active sites on the photocatalyst surface enhance the adsorption of sulfur-containing molecules, improving their redox potential compared to those with limited surface exposure. However, prolonged light exposure may cause nanoparticle aggregation, reducing catalytic stability and efficiency. To improve oxidative photocatalytic desulfurization, researchers propose strategies such as optimizing photocatalyst structures with advanced nanomaterials, introducing high-surface-area sublayers, doping with non-metals or metals, and coupling semiconductors [28,[33], [34]]. Various compounds with photocatalytic oxidation properties have been synthesized to further this goal. For example, Cam et al. removed 85 % of tetracycline by synthesizing the MnFe_2_O_4_/BiVO_4_ nanocomposite. They prevented electron-hole recombination. Also, due to the magnetic properties of the MnFe_2_O_4_ compound, the photocatalyst material can be easily recovered with an external magnet [35]. Huang et al. synthesized rGO/MnFe_2_O_4_ nanocomposite, which eliminated 83 % of methylene blue (MB). By adding rGO to the composition, it reduced MnFe_2_O_4_ particle size and increased light absorption [36]. Thaba et al. removed 98 % of methylene blue (MB) by synthesizing Fe_2_O_3_/FeVO_4_. The combination of Fe_2_O_3_ and FeVO_4_ improves photocatalytic performance by creating intermediate levels [37]. Niu et al. obtained 96.32 % tetracycline, 92.85 % sulfamethoxazole, 95.92 % ciprofloxacin (ciprofloxacin), and 97.17 % Cr (VI) removal efficiency with F–F@ FeVO_4_/ZnCo_2_O_4_ nanocomposite synthesis [38]. Rezvani et al. synthesized a new PMo11 C d@MnFe_2_O_4_ nanocatalyst and used it in the catalytic oxidative desulfurization (CODS) process. They performed the desulfurization process with an average efficiency of 95 % after five runs [39].

There are various methods for synthesizing photocatalyst materials, including sonochemical, sol-gel, hydrothermal, solvothermal, CVD (chemical vapor deposition), and PVD (physical vapor deposition), among others. Since the improvement of the crystal structure is a key factor influencing the photocatalytic process, the hydrothermal and solvothermal methods are particularly suitable for synthesizing photocatalysts with diverse, pure structures and appropriate particle size distributions. Additionally, these methods offer high biocompatibility with the environment [40].

In this research, to improve the photocatalytic efficiency in the removal of pollutants from the environment, for the first time, MnFe_2_O_4_/FeVO_4_/modified zeolite nanocomposite was synthesized by a simple and controllable two-step hydrothermal-ultrasonic method and characterized by XRD, EDS, FESEM, TEM, BET, and DRS analyses. To evaluate the photocatalytic performance of the synthesized nanocomposite, a solution of 800 ppm benzothiophene and 10 ppm methylene blue (MB) was used as pollutants. Research indicates that modified zeolite adsorbs more pollutants, together with MnFe_2_O_4_ due to its magnetic properties that collect the photocatalyst following the process, and FeVO_4_ also contains a high percentage of desulfurization potential. Therefore, by creating a multi-component nanocomposite of these compounds, efficiency is expected to improve.

## 2 Experimental

### 2.1 Materials and measurements

Manganese nitrate tetrahydrate (Mn)NO_3_(_2_.4H_2_O ≥ 99.00 %), iron (III) nitrate nonahydrate (Fe(NO_3_)_3_.9H2O ≥ 99.95 %), ammonium metavanadate (NH_4_VO_3_ ≥99.00 %), hydrazine (N_2_H_4_ ≥99.00 %), ammonium hydroxide (NH_4_OH), sodium hydroxide (NaOH ≥99.00 %), cetyltrimethylammonium bromide (C_19_H_42_BrN), methylene blue (C_16_H_18_ClN_3_S), benzothiophene (BT), n-hexane (C6H14), acetonitrile (C_2_H_3_N ≥ 99.80 %), and ethanol (C_2_H_6_O) were purchased from Sigma-Aldrich and Merck. All chemicals used were highly purified and were employed without further purification. Diffusion reflectance spectroscopy (DRS) was performed via a Shimadzu UV3600Iplus. Using a Zeiss Sigma300-HV instrument, FESEM (field emission scanning electron microscope) images were recorded. A Philips-XPertPro instrument was used to examine XRD patterns, which were filtered with Ni-filters and then emitted with Cu Kα radiation. Magna-IR devices-specifically a Nicolet 550 spectrometer-were utilized to analyze FT-IR (Fourier transform infrared). The resolution range was 0.125 cm on KBr tablets covering 400–4000 cm⁻^1^. The experiment was carried out through an X-ray scattering apparatus from Philips XL30 for energy dispersive spectroscopy (EDS). The BET (Brunauer-Emmett-Teller) surface area of the sample and the N_2_ adsorption/desorption isotherms were measured using Microtrac's BELSORP MINI X device. Sulfur content was measured with a sulfur analyzer in the oil model with a Bruker SCION 436 GC/MS device. Eventually, the remaining pollutant concentration in the samples was determined using the Shimadzu UV–1650 P C UV–Vis Spectrophotometer at the pollutant maximum wavelength.

### 2.2 Synthesis of MnFe_2_O_4_/FeVO_4_

The synthesis of the MnFe_2_O_4_/FeVO_4_ composite was carried out through a series of well-defined steps. First, 3 ml of hydrazine was diluted in 10 ml of distilled water and stirred for 5 min to ensure a homogeneous solution. Then, 1 mmol of manganese salt was dissolved in 20 ml of distilled water. This solution was then transferred to the hydrazine solution and mixed for 10 min to facilitate the reaction. Following this, 3 mmol of iron salt was dissolved in 20 ml of distilled water, added to the previous mixture, and stirred for an additional 10 min to ensure thorough mixing. Next, 1 mmol of vanadium salt was dissolved in 20 ml of distilled water and added to the mixture. This was stirred for an additional 10 min, maintaining room temperature throughout the process. The final solution was transferred to an autoclave and subjected to heating in an oven at 180 °C for 12 h. After the heating process, the solution was carefully removed from the autoclave. The resulting precipitate was washed with distilled water and ethanol to remove any unreacted materials and impurities. Finally, the precipitate was dried at 60 °C for 5 h, resulting in sample H3. The detailed reaction conditions and parameters are summarized in Table 1, which provides further insights into the synthesis process. This methodical approach ensures the successful formation of the desired composite with high purity and structural integrity.

**Table 1:** Reaction conditions for the synthesis of MnFe_2_O_4_/FeVO_4_ nanostructures.

Sample	Mn	Fe	V	N_2_H_4_	NH_3_	NaOH	product
H1	1	2	–	3	–	–	MnFe_2_O_4_/MnO_2_
H2	–	1	1	3	–	–	Fe_0.5_V_3.5_O_8_/FeO_2_
H3	1	3	1	3	–	–	MnFe_2_O_4_/FeVO_4_
H4	1	3	1	–	3	–	Amorph
H5	1	3	1	–	–	3	MnFeO_3_/Fe_2_VO_4_/Mn_2_V_2_O_7_

### 2.3 Zeolite modified by CTAB

The amount of CTAB surfactant was prepared 10 times its CMC concentration and mixed with zeolite at a weight ratio of 1:3 for 12 h. Afterward, it was washed with distilled water and dried at 60 °C (sample H6). CMC or critical micelle concentration, is identified as a concentration of surfactants at which molecules begin to form micelles.

### 2.4 Synthesis of MnFe_2_O_4_/FeVO_4_/modified zeolite

First, 0.401 g of MnFe_2_O_4_/FeVO_4_ was dispersed in 10 ml of distilled water using ultrasonic waves. 2.74 g of modified zeolite was dispersed in 10 ml of distilled water and added to the previous solution and ultrasonicated at 100 W for 15 min. The obtained nanocomposite was dried at 60 °C in an oven (sample HS7). Two samples were synthesized with ratios of 1:1 and 1:2 for MnFe_2_O_4_/FeVO_4_: modified zeolite and were named sample HS7 and sample HS8, respectively.

### 2.5 PODS evaluation

#### 2.5.1 Methylene blue degradation

The first step was to prepare a 100 ml aqueous solution of methylene blue (MB) containing a concentration of 10 ppm. Following the addition of 50 mg of the synthesized photocatalyst, it took 45 min of stirring in the dark on a stirrer until adsorption-desorption equilibrium was reached. The suspension was then exposed to visible light for 45 min using a 400 W Osram lamp. After 15 min of irradiating the solution, 5 ml was removed, and centrifugation was conducted to separate the photocatalyst. It is important to mention that the samples containing MnFe_2_O_4_ were easily separated from the suspension using a magnet due to the magnetic property of this substance and there was no need to centrifuge. The concentration of the aqueous solution was then determined through a spectrophotometer with a maximum adsorption wavelength of methylene blue (MB) (664 nm). Centrifugation was used to separate the photocatalyst from the solution. This was followed by several washings with distilled water and drying at 70 °C for a few minutes. The photodegradation efficiency (*R*) of MB was calculated as follows:

(1) $$R=\frac{\left(C_{0}-C_{1}\right)}{C_{0}}\times 100$$

where *C*_0_ and *C*_1_ are the initial concentration and the residual concentration of methylene blue solution at reaction time *t*, respectively.

#### 2.5.2 Benzothiophene desulfurization

To check the photocatalytic performance of the synthesized samples, an 800 ppm benzothiophene solution was prepared. Then 0.05 g of synthesized samples were weighed and poured into 100 ml of standard solution. They enter the reactor and the aeration process occurs. Aeration is performed in the photocatalytic process to facilitate and enhance photoreactions and provide the necessary energy for electron-hole pair formation. Afterward, it is placed in a dark environment for 45 min. This is so that the sulfur adsorption process is carried out on the photocatalyst surface. Then it is exposed to visible light for 45 min and sampling is done at the end of the process. Following separation in a centrifuge, it is washed in acetonitrile. Finally, using GC-MS analysis, the amount of sulfur in the solution is calculated, while the percentage of sulfur removal is measured using the following formula.

(2) $$R=\frac{\left(S_{0}-S_{1}\right)}{S_{0}}\times 100$$

where S_0_ and S are the sulfur content before and after light irradiation. A similar method was used to investigate the desulfurization capability of the synthesized photocatalyst from diesel fuel as a real fuel. The results of MB degradation and desulfurization are shown in Table 2. To conduct the catalyst recycling test, at the end of the photocatalytic process, the photocatalyst (MnFe_2_O_4_/FeVO_4_/modified zeolite (2:1)) was separated using a magnet, then washed with ethanol and dried at 70 °C for 3 h. Subsequently, Then, the photocatalytic reaction of pollutant removal (methylene blue solution, benzothiophene solution, and diesel fuel) was performed in the presence of recycled catalyst.

**Table 2:** Photocatalytic efficiency of synthesized products.

Sample	Product	MB degradation%	Desulfurization%
H1	MnFe_2_O_4_/MnO_2_	66	67
H2	Fe_0.5_V_3.5_O_8_/FeO_2_	60	28
H3	MnFe_2_O_4_/FeVO_4_	67	68
H4	Amorph	58	45
H5	MnFeO_3_/Fe_2_VO_4_/Mn_2_V_2_O_7_	64	22
HS7	MnFe_2_O_4_/FeVO_4_/modified zeolite	84	83
HS8	MnFe_2_O_4_/FeVO_4_/modified zeolite	93	100

## 3 Results and discussion

### 3.1 Characterization

#### 3.1.1 XRD studies

**Fig. 1:**
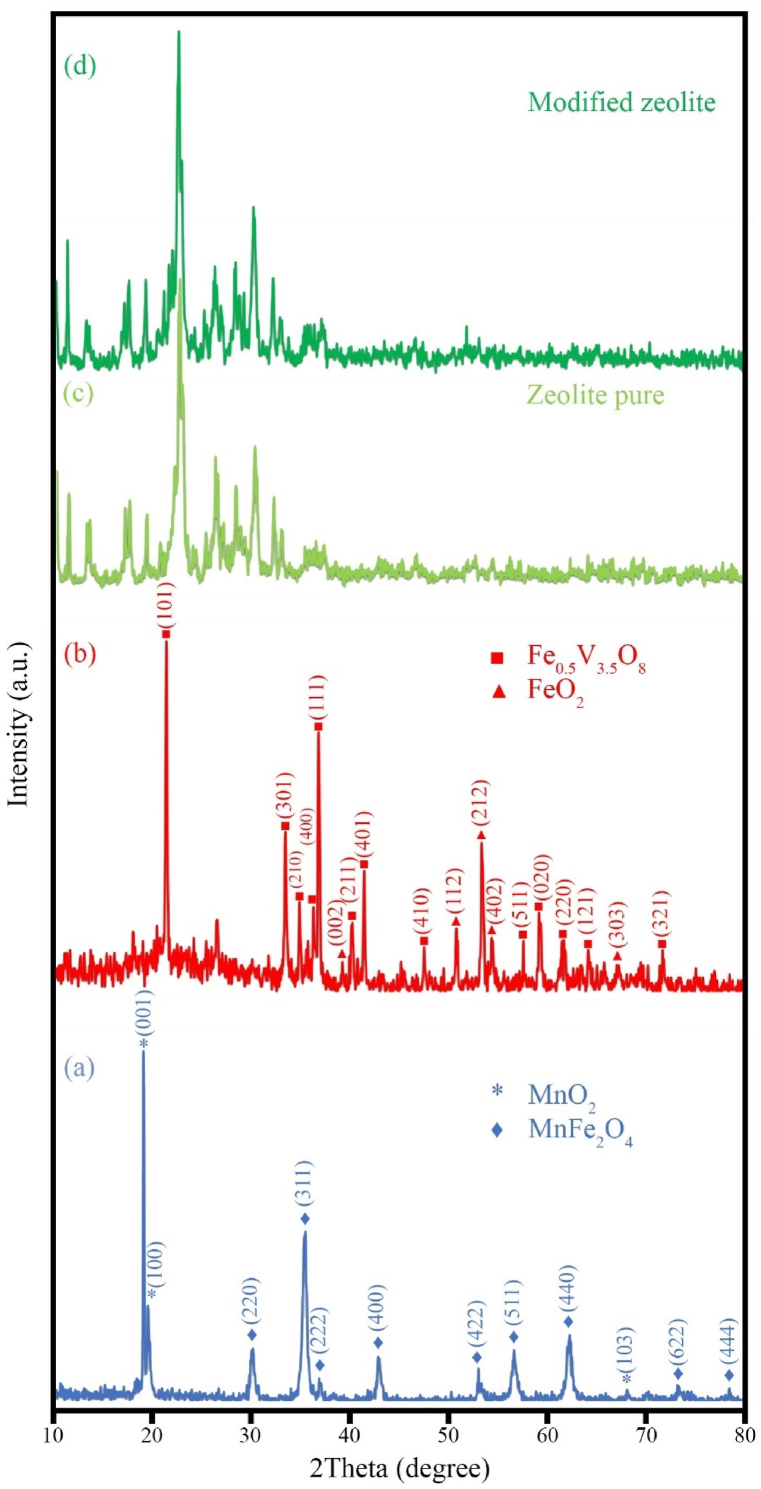
XRD patterns of the as-synthesized products (a) sample H1 (MnO_2_/MnFe_2_O_4_), (b) sample H2 (FeO_2_/Fe_0.5_V_3.5_O_8_), (c) pure zeolite, and (d) modified zeolite.

**Fig. 2:**
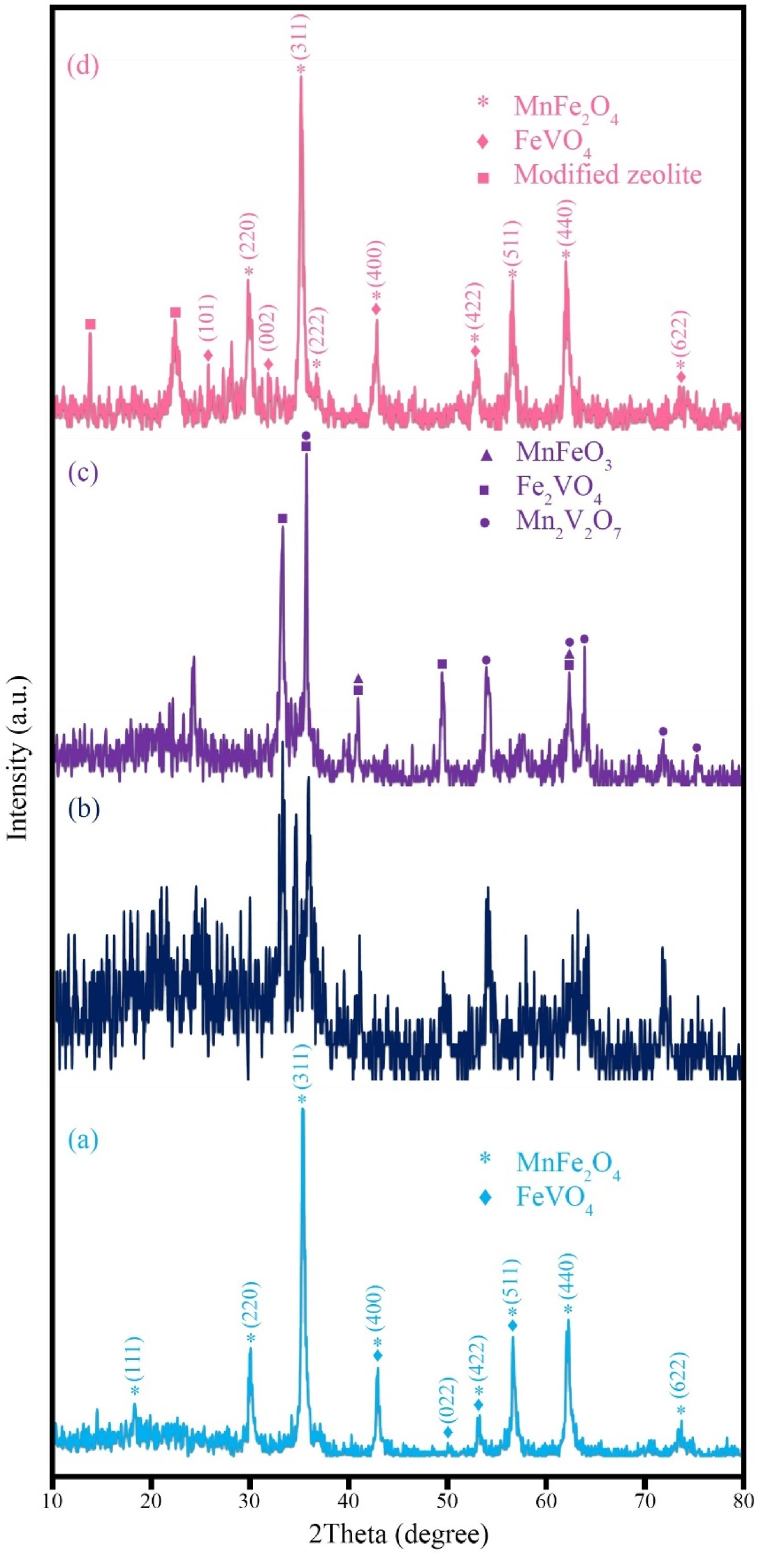
XRD patterns of the as-synthesized products (a) sample H3 (MnFe_2_O_4_/FeVO_4_), (b) sample H4 (Amorph), (c) sample H5 (MnFeO_3_/Fe_2_VO_4_/Mn_2_V_2_O_7_), (d) sample HS8 (MnFe_2_O_4_/FeVO_4_/modified zeolite (1:1)).

The XRD patterns of the synthesized samples are shown in Fig. 1, Fig. 2. Fig. 1a corresponds to sample H1 synthesized using 3 mL of hydrazine along with Mn and Fe salts. Sample H1 consists of two chemical compounds: MnFe_2_O_4_ with a cubic crystal structure (JCPDS No. 00-010-0319 and lattice parameters a = 8.4990 Å), as well as MnO_2_ with a hexagonal crystal structure (JCPDS No. 96-900-9112 and lattice parameters a = 3.3400 Å, b = 3.3400 Å, and c = 4.6800 Å). The pattern of sample H2 is shown in Fig. 1b, which was synthesized using Fe and V salts along with 3 ml of hydrazine. The sample H2 consists of two chemical compounds: Fe_0.5_V_3.5_O_8_, which has an orthorhombic crystal structure (JCPDS No. 96-900-0060), with lattice parameters a = 9.9700 Å, b = 3.0300 Å, and c = 4.5400 Å, and FeO₂, which also has an orthorhombic structure (JCPDS No. 96-901-6407), with lattice parameters a = 9.9600 Å, b = 3.0230 Å, and c = 4.6050 Å. The results indicate that MnO_2_ and FeO_2_ are present as by-products in the sample. The presence of both compounds in the sample is likely due to the oxygen content in the solution. Oxygen can significantly affect the nucleation process, and it appears that the sample was not synthesized in its pure form. Fig. 1c and d shows raw zeolite samples and modified zeolite samples. It is evident that in the modified zeolite, the intensity of the peaks has changed. Fig. 2 displays the XRD patterns of samples H3, H4, H5, and HS8. These samples were synthesized using different -OH^-^ sources. Specifically, Fig. 2a corresponds to sample H3, which was synthesized using 3 ml of hydrazine. Two chemical compounds are formed in sample H3: MnFe_2_O_4_ with a cubic crystal structure (JCPDS No. 00-010-0319 and lattice parameters a = 8.4990 Å), and FeVO_4_ with an orthorhombic crystal structure (JCPDS No. 00-015-0029-00 and lattice parameters a = 4.4900 Å, b = 5.5300 Å, and c = 4.9000 Å). The sample was carefully prepared with precise control of excess manganese and iron to avoid impurities that might affect the crystal and chemical structure. The results reveal a distinct composition of iron vanadate oxide compared to the sample H2. The compositional difference results in distinct lattice parameters, leading to the unique crystal structure of the sample. Fig. 2b illustrates the XRD pattern of sample H4, synthesized using 3 mL of ammonia as the -OH- source, which displays a quasi-crystalline structure. In contrast, Fig. 2c depicts the XRD pattern of sample H5, synthesized with 3 mL of NaOH, showcasing an improved crystal structure compared to sample H4. However, as observed, various compounds, including MnFeO_3_ with a cubic crystal structure (JCPDS No. 01-076-0076 and lattice parameters a = 9.3650 Å), and Fe_2_VO_4_ with a cubic crystal structure (JCPDS No. 96-591-0013 and lattice parameters: a = 8.3000 Å, b = 8.3000 Å, and c = 8.3000 Å), have been synthesized. Additionally, Mn_2_V_2_O_7_ with a monoclinic crystal structure (JCPDS No. 01-075-1224 and lattice parameters: a = 6.1790 Å, b = 8.7190 Å, and c = 4.9660 Å) has formed. Notably, the change in the -OH^-^ source alters the synthesis conditions and enables precise control over the composition and structure of the synthesized materials. The controlled and uniform release of -OH^-^ by hydrazine contributes to the formation of a thermodynamically stable pure product with an improved crystal structure. In Fig. 2d, a ternary nanocomposite (sample HS8) with the chemical compositions MnFe₂O₄ (cubic crystal structure, JCPDS No. 00-010-0319, lattice parameter a = 8.4990 Å) and FeVO₄ (orthorhombic crystal structure, JCPDS No. 0294-015-00, with lattice parameters a = 4.4900 Å, b = 5.5300 Å, and c = 4.9000 Å) was synthesized in the vicinity of the modified zeolite.

#### 3.1.2 FT-IR studies

**Fig. 3:**
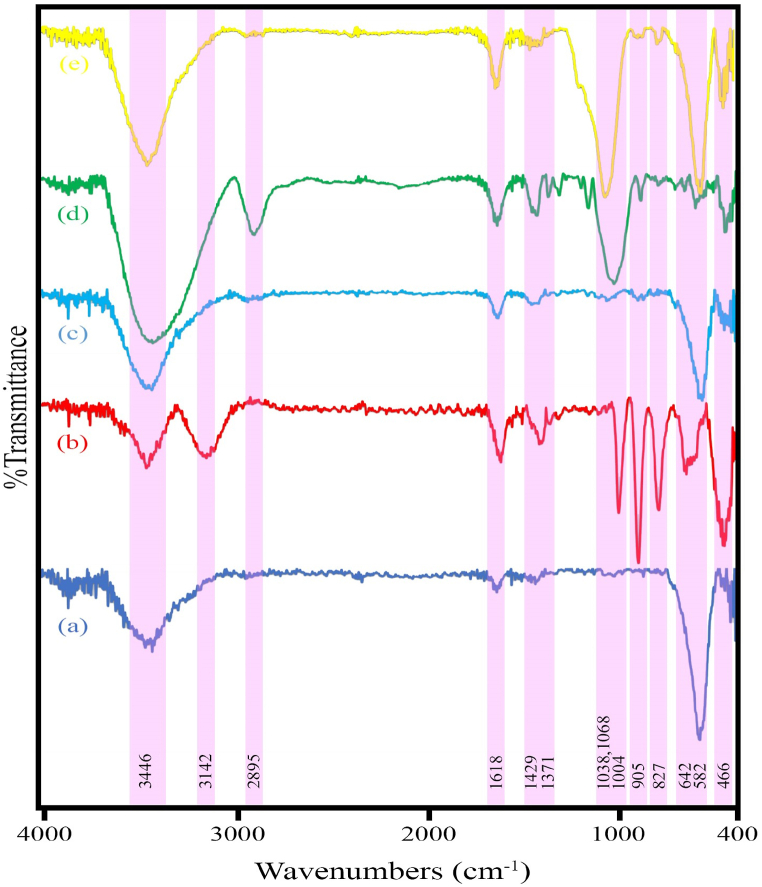
FT-IR spectra of the as-synthesized products (a) sample H1 (MnO_2_/MnFe_2_O_4_), (b) sample H2 (FeO_2_/Fe_0.5_V_3.5_O_8_), (c) sample H3 (MnFe_2_O_4_/FeVO_4_), (d) sample H6 (modified zeolite), (e) sample HS7 (MnFe_2_O_4_/FeVO_4_/modified zeolite (1:1)).

Fig. 3a–e shows the FTIR spectra of samples H1 (MnO_2_/MnFe_2_O_4_), H2 (FeO_2_/Fe_0.5_V_3.5_O_8_), H3 (MnFe_2_O_4_/FeVO_4_), H6 (modified zeolite), and HS7 (MnFe_2_O_4_/FeVO_4_/modified zeolite (1:1)), respectively. In all spectra, the peaks at 3418 and 1634 cm^−1^ correspond to strong and broad bonds, representing the symmetric and asymmetric stretching of the O-H bond due to water adsorption, respectively. Additionally, the peaks at 582 cm^−1^ correspond to the Mn-O stretching bond, while those at 642 and 466 cm^−1^ correspond to the Fe-O stretching and bending bonds, respectively. The peaks at 827, 905, and 1004 cm^−1^ indicate the coupled vibrations of V-O and V-O-V, associated with the short stretching of the vanadyl bond. Fig. 3d represents the zeolite modified with CTAB (sample H6). The peaks at 2895, 1429, and 1371 cm^−1^ are attributed to the C-H bond, resulting from the modification of the zeolite surface by CTAB. Furthermore, the peaks at 1068 and 1038 cm^−1^ arise from the asymmetric stretching vibration of MO_4_ (where M = Si, Al). Finally, Fig. 3e illustrates the spectrum of sample HS7, encompassing all the chemical bonds [[37], [38], [39],^41^].

#### 3.1.3 SEM and TEM studies

**Fig. 4:**
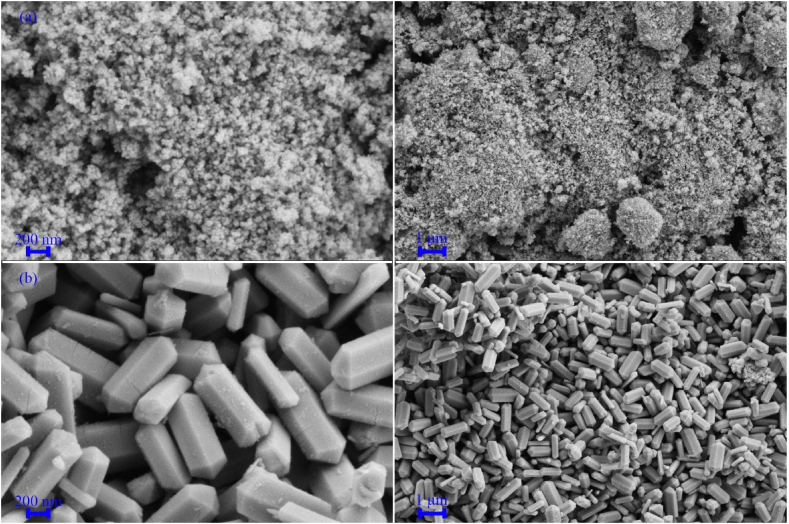
FESEM images of the as-synthesized products (a) sample H1 (MnO_2_/MnFe_2_O_4_) and (b) sample H2 (FeO_2_/Fe_0.5_V_3.5_O_8_).

**Fig. 5:**
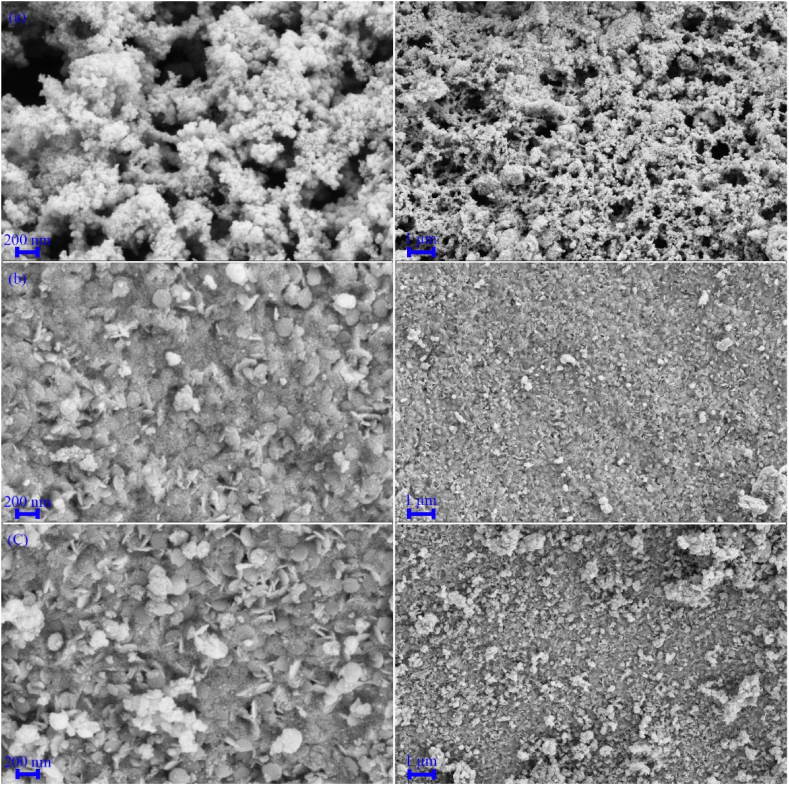
FESEM images of the as-synthesized products (a) sample H3 (MnFe_2_O_4_/FeVO_4_), (b) sample H4 (amorph), (c) sample H5 (MnFeO_3_/Fe_2_VO_4_/Mn_2_V_2_O_7_).

**Fig. 6:**
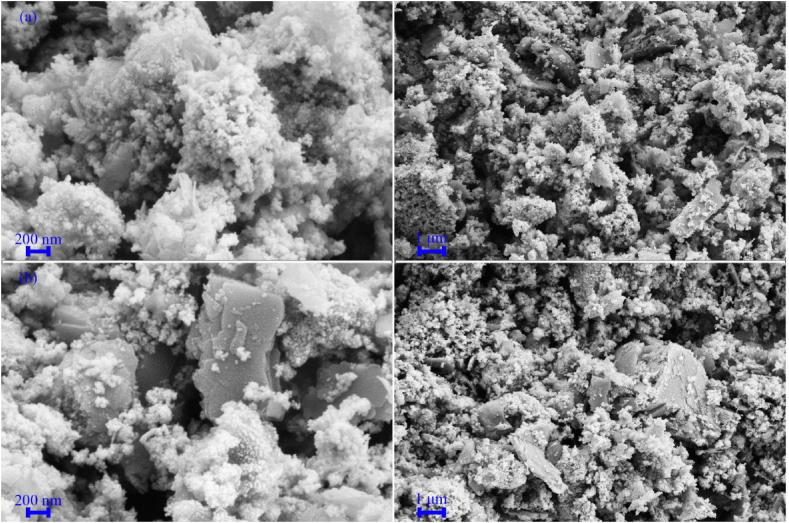
FESEM images of the as-synthesized products (a) sample HS7 (MnFe_2_O_4_/FeVO_4_/modified zeolite (1:1)), and (b) sample HS8 (MnFe_2_O_4_/FeVO_4_/modified zeolite (2:1)).

**Fig. 7:**
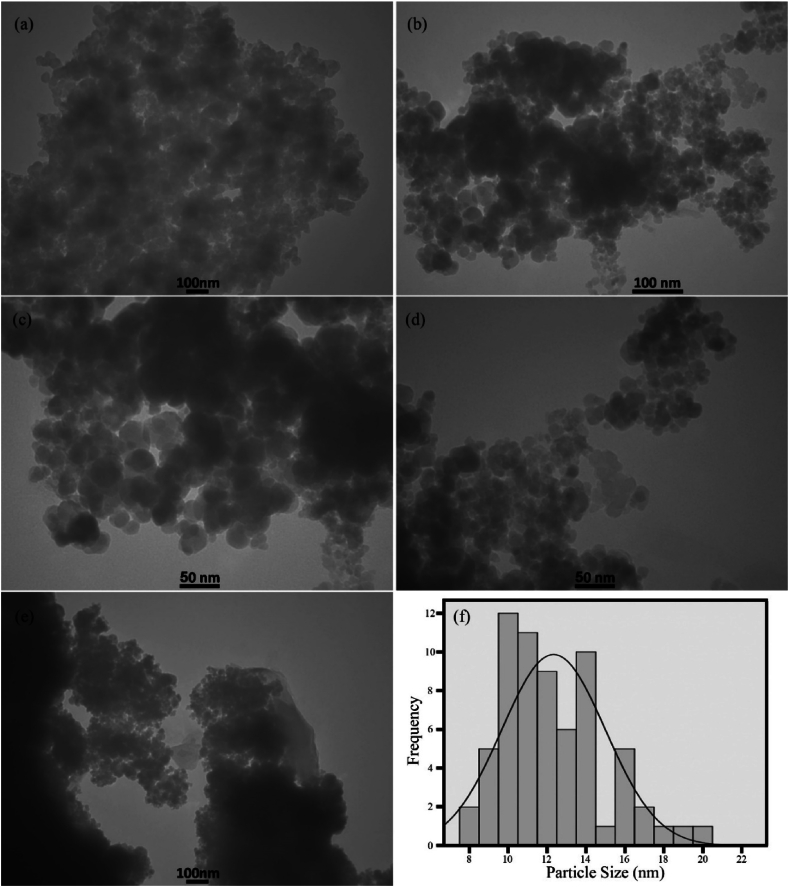
(a–e) TEM images of sample HS8 ((MnFe_2_O_4_/FeVO_4_/Modified Zeolite (2:1)) at different magnifications and (f) Image of particle size distribution of sample HS8 (MnFe_2_O_4_/FeVO_4_/modified zeolite (2:1)).

Fig. 4, Fig. 5, Fig. 6 show FESEM images of the synthesized samples. Fig. 4a, which corresponds to sample H1, shows spherical MnFe_2_O_4_ particles with a size of approximately 25 nm next to some MnO_2_ structures. These MnO_2_ nanoparticles self-assemble into spherical structures with a diameter of 2 μm. Fig. 4b displays FESEM images of Fe_0.5_V_3.5_O_8_ polyhedral microstructures with a diameter of 200 nm and a length of about 1 μm. Fig. 5a corresponds to sample H3, where the product contains MnFe_2_O_4_/FeVO_4_ nanoparticles uniformly distributed. Notably, the presence of MnFe_2_O_4_ leads to the fragmentation of Fe_0.5_V_3.5_O_8_ microstructures into FeVO_4_ nanostructures. Fig. 5b and c illustrate the synthesized composites H5 and H6, respectively. In these composites, ammonia and sodium hydroxide were used as substitutes for hydrazine to generate hydroxide ions. The results indicate the presence of larger particles and agglomeration effects, suggesting that hydrazine is superior to the other sources of OH^−^. Adjusting the concentration of hydroxide ions influences both the nucleation and growth rates, which in turn affects particle size [42]. While ammonia and sodium hydroxide are commonly employed in the synthesis of metal oxides due to their rapid release of OH^−^ into the environment, hydrazine provides a more controlled and uniform introduction of hydroxide ions, making it the optimal choice for product synthesis. The FESEM images of samples HS7 and HS8, which were synthesized with ratios of 1:1 and 1:2 for MnFe_2_O_4_/FeVO_4_: modified zeolite, are presented in Fig. 6a and b, respectively. As can be seen in Fig. 6, MnFe_2_O_4_ and FeVO_4_ nanoparticles with spherical morphology are well distributed on the modified zeolite surface. By using zeolite, finer particles are produced because suitable places for nucleation are provided. The number of layers aligns with expectations based on Fig. 6b, where the nanoparticles are deposited on the modified zeolite layers. Fig. 7a–e shows TEM images of MnFe_2_O_4_/FeVO_4_/modified zeolite (2:1) nanocomposite at different magnifications. Two distinct particle sizes are visible, larger particles corresponding to FeVO_4_ and smaller particles corresponding to MnFe_2_O_4_, and this result is compatible with the XRD results. Additionally, the layered structure of the modified zeolite is visible. Furthermore, Fig. 7f provides valuable information regarding particle size distribution, with an average particle size of approximately 12.5 nm.

#### 3.1.4 EDS studies

**Fig. 8:**
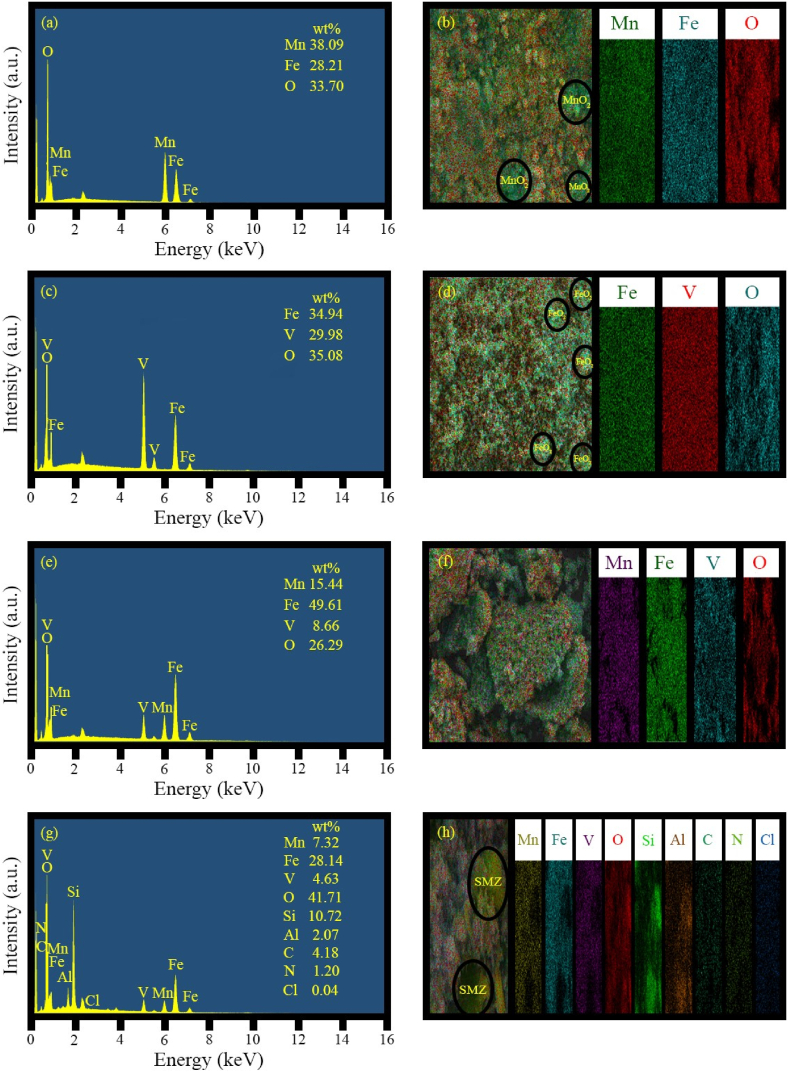
EDS and EDS mapping spectra of the as-synthesized products (a) and (b) sample H1 (MnO_2_/MnFe_2_O_4_), (c) and (d) sample H2 (FeO_2_/Fe_0.5_V_3.5_O_8_), (e) and (f) sample H3 (MnFe_2_O_4_/FeVO_4_), (g) and (h) sample HS8 (MnFe_2_O_4_/FeVO_4_/modified zeolite (2:1)).

Fig. 8a corresponds to the EDS spectrum of MnFe_2_O_4_ (sample H1). As can be seen, only Mn, Fe, and O elements are present, and no other impurities are visible. Fig. 8b, which shows the elemental distribution for sample H1, confirms good distribution in most parts and supports the presence of MnFe_2_O_4_ composition. Moreover, in some regions, only Mn and O are detected, indicating the formation of MnO_2_ by-product, which is consistent with the XRD results. In Fig. 8c, only Fe, V, and O elements are present, and no other impurities are visible. According to Fig. 8d, which shows the element distribution for the sample H2, there is good distribution in most parts, confirming the presence of Fe_0.5_V_3.5_O_8_ composition. Furthermore, in some regions, only Fe and O are detected, indicating the formation of FeO_2_, consistent with the XRD results. Fig. 8e and f shows that the Mn, Fe, V, and O elements in the MnFe₂O₄/FeVO₄ nanocomposite (sample H3) are well-distributed and exhibit significant uniformity, confirming the successful synthesis of the nanocomposite. Additionally, Fig. 8g and h demonstrate the proper distribution of the nanocomposite on the modified zeolite layers in sample HS8.

#### 3.1.5 DRS studies

**Fig. 9:**
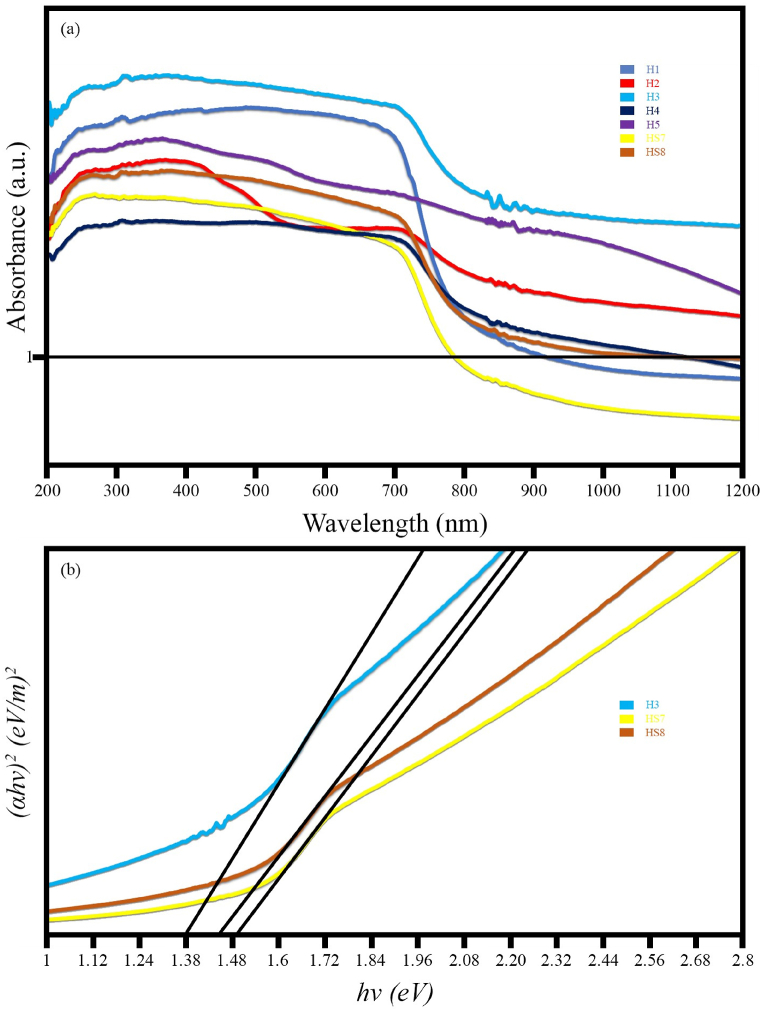
(a) UV–Vis DRS curves and (b) the plots of (αhν)^2^ against hν of the as-synthesized products.

Fig. 9a displays the UV–Vis absorption spectra for the synthesized samples. Sample H3 exhibits the highest absorption in both visible and UV regions compared to the other synthesized samples. MnFe_2_O_4_ shows acceptable absorption in both visible and ultraviolet regions. However, Fe_0.5_V_3.5_O_8_ demonstrates significant absorption in the ultraviolet region, while its absorption decreases in the visible region. These results indicate the formation of a nanocomposite with broad light absorption properties when combining MnFe_2_O_4_ and FeVO_4_. Samples H4 and H5 exhibit lower absorption than sample H3. The FESEM results confirm the well-distributed MnFe_2_O_4_/FeVO_4_ nanoparticles in the sample H3. In the samples synthesized with sodium hydroxide and ammonia, agglomeration effects are observed, and these results can be justified. With the addition of modified zeolite, light absorption decreases compared to the H3 nanocomposite. However, zeolite itself does not absorb light very well. Nevertheless, due to the significant amount of pollutant adsorption, the substantial effect of zeolite on degradation efficiency is promising and warrants further investigation. The bandgaps for samples H3 (MnFe_2_O_4_/FeVO_4_), HS7 (MnFe_2_O_4_/FeVO_4_/modified zeolite (1:1)), and HS8 (MnFe_2_O_4_/FeVO_4_/modified zeolite (2:1)) are presented in Fig. 9b. As can be seen, with the addition of zeolite, the bandgap increases by 0.1 eV (from 1.38 eV to 1.48 eV), which is an acceptable result.

#### 3.1.6 BET analysis

**Fig. 10:**
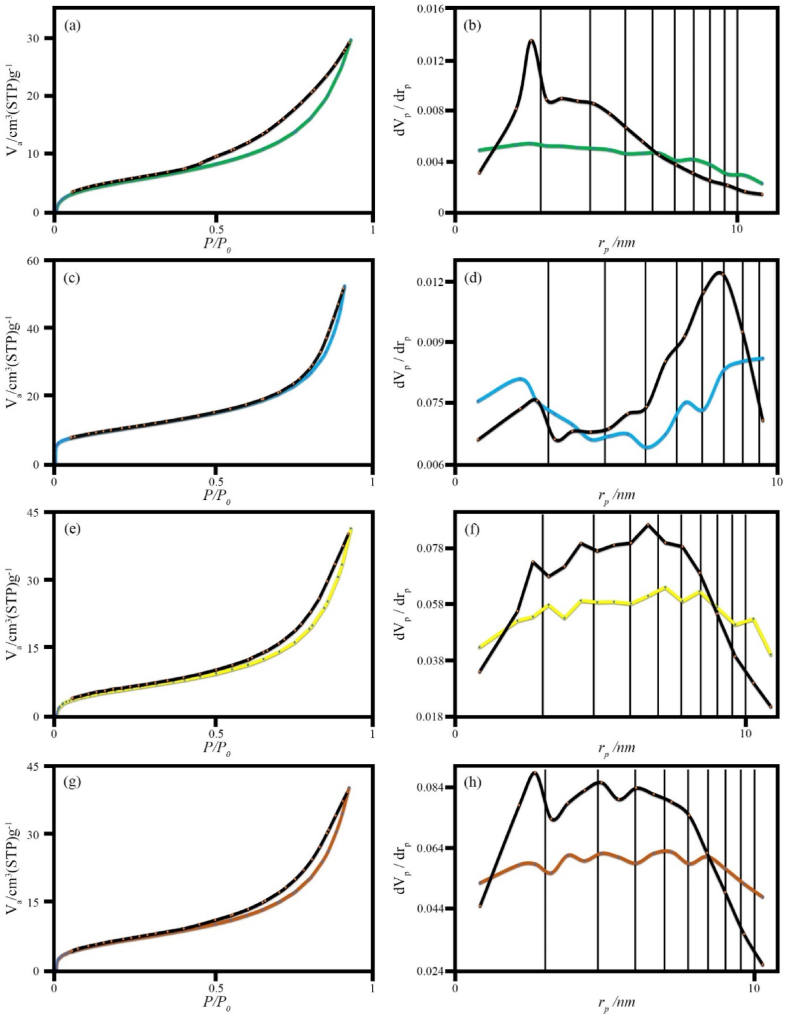
N_2_ adsorption/desorption isotherms and BJH pore size distribution of the as-synthesized products (a) and (b) sample H6 (modified zeolite), (c) and (d) sample H3 (MnFe_2_O_4_/FeVO_4_), (e) and (f) sample HS7 (MnFe_2_O_4_/FeVO_4_/modified zeolite (1:1)), (g) and (h) sample HS8 (MnFe_2_O_4_/FeVO_4_/modified zeolite (2:1)).

The results of BET, BJH and Ads.-Des. have been shown in Fig. 10a–h. In all samples, the adsorption and desorption isotherms are porous and mesoporous. The surface area of sample H3 is 35 m^2^/g and the average pore diameter is 9.23 nm, which is broader than that of modified zeolite (sample H6), which is 17 m^2^/g and 1.85 nm. Nanocomposite H3 and modified zeolite (sample HS8) have a surface area of 22 m^2^/g and average pore diameters of 5.29 nm, as expected.

### 3.2 Pollutant removal

#### 3.2.1 Methylene blue degradation

The presence and absence of a photocatalyst were investigated to understand their effect on the process of pollutant degradation. It was observed that in the absence of a photocatalyst, almost no degradation occurs. However, in the presence of a photocatalyst, significant degradation is observed. Notably, sample HS8 exhibits the highest efficiency. On the other hand, sample H6, which is modified zeolite, does not participate in the degradation process but acts as an adsorbent, confirming this behavior. When comparing sample HS8 with ample HS7, the increase in efficiency can be attributed to the higher utilization of photocatalysts. According to the obtained results, it was observed that the samples with modified zeolite exhibit higher efficiency compared to the samples without modified zeolite, with a significant increase in efficiency from 67 % to 93 %. This highlights the effect of modified zeolite. Among the synthesized products, sample H3 which contains MnFe_2_O_4_ and FeVO_4_, demonstrates the best efficiency (approximately 67 %), surpassing samples H2 and H1. It can be inferred that a synergistic effect occurs due to the formation of this nanocomposite. Additionally, the decomposition of Fe_0.5_V_3.5_O_8_ microstructures into FeVO_4_ nanostructures also contributes to this synergism (as seen in FESEM images). Despite the results from BET and DRS not showing an increase, the zeolite's impact is evident, possibly altering adsorption kinetics, an aspect worth further investigation.

##### 3.2.1.1 Effect of pH

**Fig. 11:**
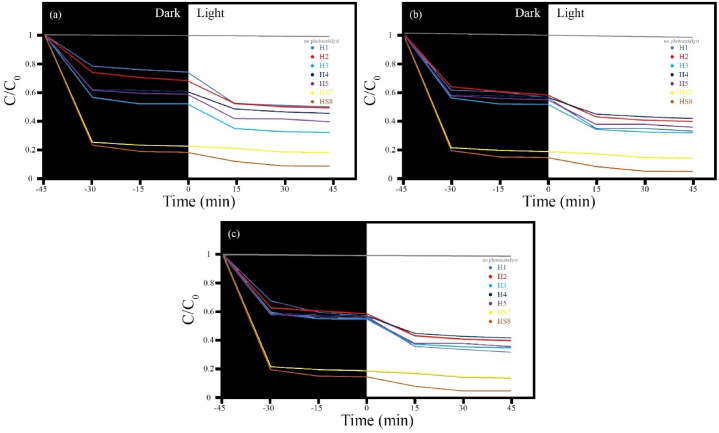
The effect of pH values of pollutant solution on photocatalytic degradation using synthesized samples over time for 50 mg of different catalysts in 100 ml of MB solution at ambient temperature and pH = 4 (a), pH = 7 (b), pH = 9 (c).

The initial pH of the methylene blue (MB) solution is a critical parameter in the photocatalytic process. To determine the optimal pH for methylene blue (MB), while keeping further influential parameters constant, the effect of initial pH was investigated, and the results are shown in Fig. 11. As can be seen, pollutant degradation speed increases with higher solution pH. The ideal photocatalytic degradation rate occurs at pH = 7, which provides optimal conditions for the reaction (Fig. 11a). On the other hand, at pHs 4 and 9 (Fig. 11b and c, respectively), complexes are formed that are unsuitable for pollutant degradation due to new reactions involving OH and H radicals in solution.

##### 3.2.1.2 Effect of photocatalyst dose

**Fig. 12:**
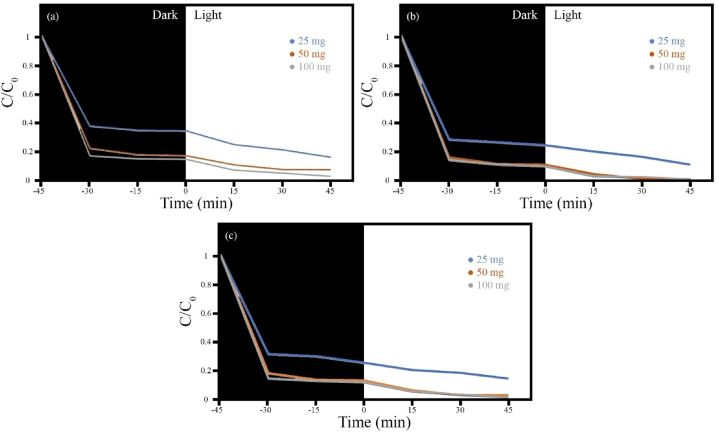
Photocatalytic degradation of MB using different doses of photocatalyst HS8 (MnFe_2_O_4_/FeVO_4_/modified zeolite (2:1)) (100 mg/L, MB solution at different pH).

While upholding additional influential factors constant, the impact of HS8 photocatalyst dosage on pollutant degradation was systematically evaluated at various pH levels (see Fig. 12). As can be seen, pollutant degradation increases with an increasing dosage at different pH values. The production of active radicals OH and O_2_^−^ and their interaction with pollutant molecules play a crucial part in the photocatalytic degradation procedure. A close correlation exists between degradation efficiency and the availability of the HS8 photocatalyst surface. Increasing the charge on the photocatalyst enhances surface accessibility, allowing for efficient light absorption and pollutant adsorption. Consequently, additional active radicals are produced during the photocatalytic operation, leading to enhanced pollutant decomposition speed.

##### 3.2.1.3 Mechanism of photocatalytic degradation

**Fig. 13:**
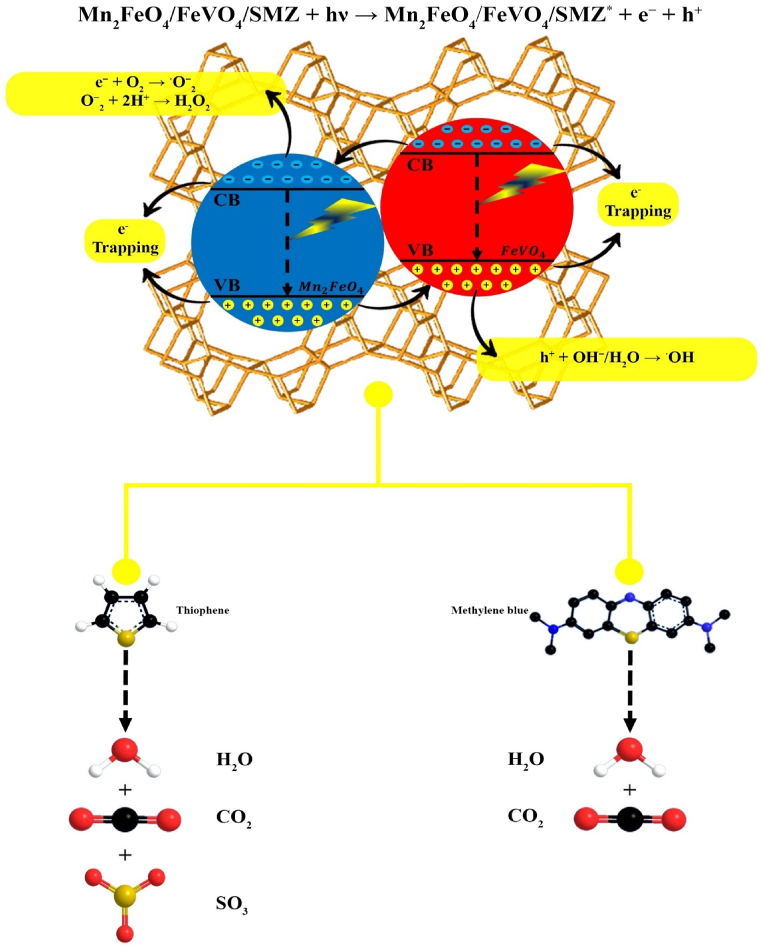
Proposed photocatalytic reaction mechanism for methylene blue decomposition and benzothiophene desulfurization by MnFe_2_O_4_/FeVO_4_/modified zeolite (2:1) nanocomposite.

Fig. 13 illustrates the conceivable mechanism of the photocatalytic reaction. Functional species, including hydroxyl radicals (^•^OH), superoxide ions (^•^O_2_^−^), and holes (h^+^), play decisive roles in the photocatalytic degradation procedure. Naturally, after UV irradiation, the holes (h^+^) constructed in the conduction band (VB) may respond with OH^−^ or H_2_O to eventually beget ^•^OH radicals. Alternatively, in the conduction band (CB), O_2_ molecules are reduced, leading to the appearance of O_2_ radicals. Notably, ^•^OH generated from h^+^ holes in the valence band significantly influences the photocatalytic process.

##### 3.2.1.4 Degradation kinetics

**Fig. 14:**
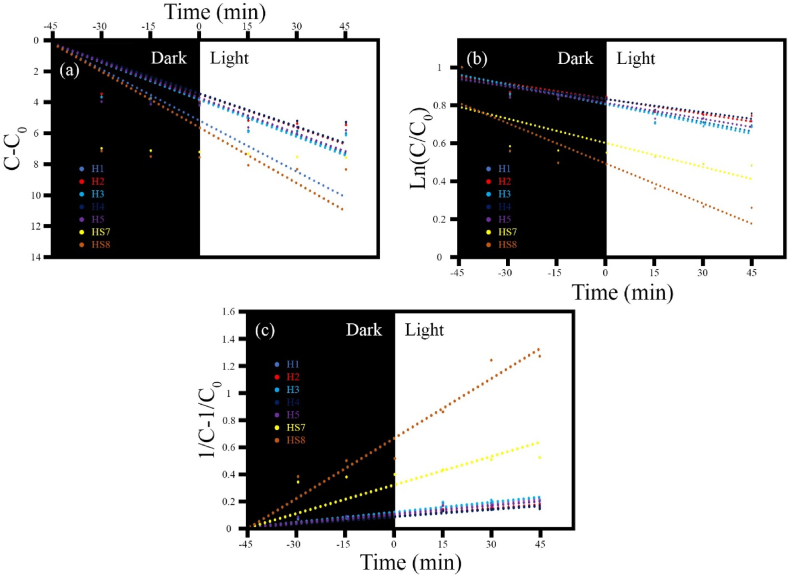
Kinetic models of (a) zero order, (b) first order, and (c) second order for the synthesized samples.

The kinetics of methylene blue (MB) degradation by the synthesized photocatalysts were scrutinized. In the degradation response, different doses of the aforementioned photocatalysts were exposed to light in a pollutant solution containing 100 mg/L MB at varying pH levels. Established on experimental data, zero-order, first-order, and second-order kinetic models were investigated and examined (Table 3). The data curves drafted from the photocatalytic procedure of the synthesized samples and their constituents are delivered in Fig. 14a–c. As shown in Table 4, the highest coefficients of determination (*R*^*2*^) in the zero-order kinetics were observed for the sample H3 (MnFe_2_O_4_/FeVO_4_), with a value of 0.9369. In the first-order kinetics of the sample H3 (MnFe_2_O_4_/FeVO_4_), the coefficient was 0.8966, while in the second-order kinetics of the sample HS8 (MnFe_2_O_4_/FeVO_4_/Modified Zeolite (2:1)), it reached an impressive value of 0.9842. The rate constant for these three values of *R*^*2*^ is 0.0916, 0.014, and 0.0149 min/1, respectively.

**Table 3:** The kinetic Equations with corresponding adjustable parameters.

Kinetic models			
Name	Equation	Parameters	References
Zero-order	$C-C_{0}=-k_{0}t$	C*, C*_*0*_*,*$K_{0}$	[46]
First-order	$\ln\left(C/C_{0}\right)=-k_{1}t$	C*, C*_*0*_*,*$K_{1}$	[46]
Second-order	$1/C-1/C_{0}=k_{2}t$	C*, C*_*0*_*,*$K_{2}$	[46]

**Table 4:** Kinetic constants of MB degradation by the synthesized photocatalysts.

Catalyst	Zero-order		First-order		Second-order	
	k_0_	R^2^	k_1_	R^2^	k_2_	R^2^
H1 (MnO_2_/MnFe_2_O_4_)	0.09	0.9332	0.014	0.8888	0.0029	0.9715
H2 (FeO_2_/Fe_0.5_V_3.5_O_8_)	0.0824	0.9245	0.012	0.8683	0.0019	0.9739
H3 (MnFe_2_O_4_/FeVO_4_)	0.0916	0.9369	0.014	0.8966	0.0025	0.9715
H4 (Amorph)	0.0814	0.8938	0.012	0.7728	0.0018	0.9488
H5 (MnFeO_3_/Fe_2_VO_4_/Mn_2_V_2_O_7_)	0.0883	0.9099	0.013	0.831	0.0022	0.9645
HS7 (MnFe_2_O_4_/FeVO_4_/modified zeolite (1:1)	0.126	0.8303	0.026	0.6994	0.0071	0.9113
HS8 (MnFe_2_O_4_/FeVO_4_/modified zeolite (2:1))	0.1374	0.8507	0.035	0.8235	0.0149	0.9842

##### 3.2.1.5 Reusability

**Fig. 15:**
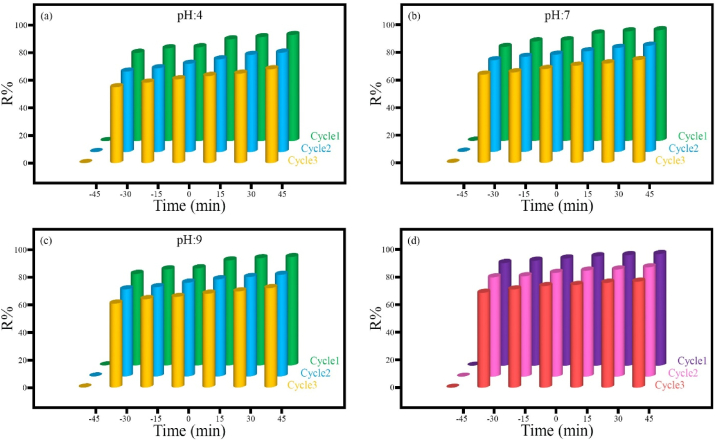
The reusability of sample HS8 (MnFe_2_O_4_/FeVO_4_/modified zeolite (2:1)) for (a–c) MB removal at an initial dye concentration of 100 mg/L at different pH and (d) benzothiophene degradation an initial concentration of 800 mg/L.

To evaluate the performance, economic efficiency and sustainability, the reusability of the sample HS8 (MnFe_2_O_4_/FeVO_4_/modified zeolite (2:1)) was investigated for both the methylene blue degradation process (Fig. 15a–c) and the benzothiophene desulfurization process (Fig. 15d). Adequate reusability and satisfactory photocatalytic performance were observed for sample HS8 over three consecutive cycles, as depicted in Fig. 15. The results indicated that after 3 cycles, the removal percentage of MB at pH = 4, pH = 7, and pH = 9 was 84 %, 92 %, and 89 %, respectively, demonstrating the high stability of sample HS8 in varying environments (Fig. 15a–c). A similar result was obtained from examining the recyclability of sample HS8 in the desulfurization of benzothiophene, so that after three cycles, the efficiency reached from 100 to 95 %, which can be a favorable result (Fig. 15d).

##### 3.2.1.6 Adsorption isotherm

To indicate the affinity between the adsorption ability of the synthesized sample and the concentration of methylene blue (MB), as well as to consider the experimental data acquired from the adsorption equilibrium, seven adsorption isotherm models were investigated: Langmuir, Freundlich, Dubinin-Radushkevich, Sips, Riedlich-Peterson, Toth, and Khan.

**Fig. 16:**
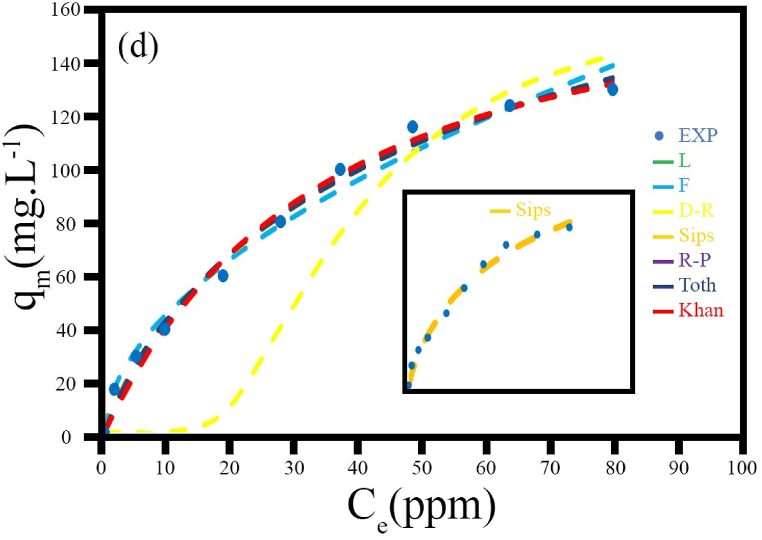
Plot of adsorption isotherms, q_e_, against C_e_, for MB removal by HS8 (MnFe_2_O_4_/FeVO_4_/modified zeolite (2:1)) at an initial dye concentration of 100 mg/L. The small figure inside shows the best-fitting model.

The equation and results of these models are shown in Fig. 16 and Table 5, Table 6. Based on the adsorption isotherms and the obtained data, the Sips model, which exhibits a maximum *R*^*2*^ value of 0.9921, was selected as the best descriptive adsorption model. The Sips model is a combination of Langmuir and Freundlich models, and it represents single-layer adsorption on a heterogeneous surface, allowing for additional interactions and leading to the multilayer effect [[43], [44], [45]].

**Table 5:** The isotherm models Equation with corresponding adjustable parameters.

Isotherm models			
Name	Non-linear Equation	Parameters	References
Langmuir	$q_{e}=\frac{q_{L}K_{L}C_{e}}{\left(1+K_{L}C_{e}\right)}$	$q_{L}$ Maximum adsorption capacity of Langmuir model (mg/g)	[44]
		$K_{L}$ Langmuir isotherm constant related to the energy of adsorption (L/g)	
Freundlich	$q_{e}=K_{F}{C_{e}}^{1/n_{F}}$	$K_{F}$ Freundlich model constant (L/g)	[44]
		$n_{F}$ Heterogeneity factor of Freundlich model (dimensionless)	
Dubinin-Radushkevich	$q_{e}=q_{D-R}exp\left[\frac{{\left(RT\;\ln\left(1+\frac{1}{C_{e}}\right)\right)}^{2}}{-2E^{2}}\right]$	$q_{D-R}$ Monolayer adsorption capacity (mg/g)	[44]
		E Characteristic adsorption energy of Dubinin–Radushkevich model (kJ/mol)	
Sips (L-F)	$q_{e}=\frac{q_{s}{\left(a_{s}C_{e}\right)}^{1/sp}}{\left({1+\left(a_{s}C_{e}\right)}^{1/\text{sp}}\right)}$	$q_{S}$ Monolayer adsorption capacity (mg/g)	[44]
		$a_{s}$ Sips constant related to the energy of adsorption (mg/L^)−1/n^	
		sp Sips isotherm exponent	
Redlich-Peterson	$q_{e}=\frac{K_{RP}C_{e}}{1+a_{RP}{C_{e}}^{\beta }}$	$K_{R-P}$ Redlich–Peterson isotherm model constant (L/g)	[44]
		$a_{R-P}$ Redlich–Peterson isotherm constant (L/mg)	
		β Redlich–Peterson isotherm exponent (0< β <1)	
Toth	$q_{e}=\frac{q_{To}C_{e}}{\left({\left[K_{To}+{C_{e}}^{tn}\right]}^{1/tn}\right)}$	$q_{To}$ Maximum adsorption capacity of Toth model (mg/g)	[44]
		$K_{To}$ Toth model constant (dimensionless)	
		tn Toth isotherm exponent (0<n≤1)	
Khan	$q_{e}=\frac{q_{K}b_{K}C_{e}}{{\left(1+b_{K}C_{e}\right)}^{a_{K}}}$	$q_{K},b_{K},a_{K}$ Khan model constants (dimensionless)	[44]

**Table 6:** The obtained isotherm constants of the MB removal by the synthesized adsorbents.

Isotherm	Parameter	HS8 (MnFe_2_O_4_/FeVO_4_/modified zeolite (2:1))
Langmuir	*q*_*L*_	200.037
	*K*_*L*_	0.026
	*R*^*2*^	0.9917
Freundlich	*K*_*F*_	13.409
	*n*_*F*_	1.862
	*R*^*2*^	0.9861
Dubinin-Radushkevich	*q*_*D-R*_	168.41
	*E*	0.976
	*R*^*2*^	0.8814
Sips	*q*_*S*_	254.26
	*sp*	0.83
	*a*_*s*_	0.031
	*R*^*2*^	0.9921
Redlich-Peterson	*K*_*R-P*_	5.98
	β	0.893
	*a*_*R-P*_	0.05
	*R*^*2*^	0.9913
Toth	*q*_*To*_	287.376
	*K*_*To*_	10.83
	*tn*	0.653
	*R*^*2*^	0.9913
Khan	*q*_*k*_	254.45
	*a*_*k*_	1.152
	*b*_*k*_	0.02
	*R*^*2*^	0.992

#### 3.2.2 Benzothiophene desulfurization

**Fig. 17:**
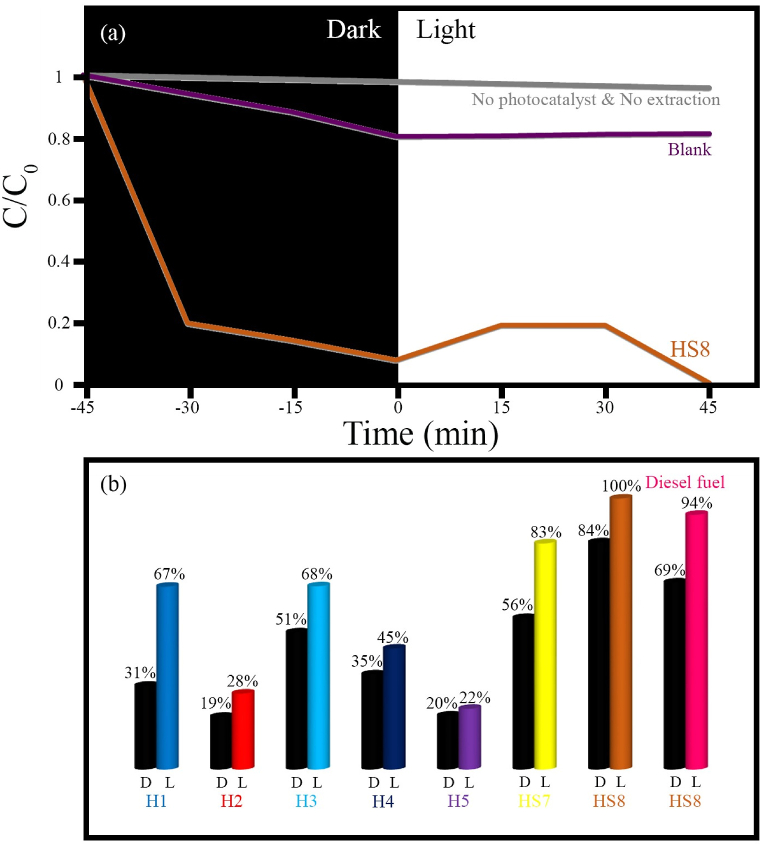
(a) Photocatalytic desulfurization of benzothiophene using sample HS8 (MnFe_2_O_4_/FeVO_4_/modified zeolite (2:1)), and (b) different amounts of photocatalytic desulfurization for the synthesized samples.

To further investigate the removal of various pollutants, the removal of pollutants from the synthesized samples onto sulfur was also evaluated and analyzed) Fig. 17). As shown in Fig. 17a, when neither a photocatalyst nor an extractant (acetonitrile) is present, the desulfurization efficiency is minimal. In the blank test, where only the extractant is used without the photocatalyst, an efficiency of 21 % is achieved. Notably, sample HS8 (MnFe₂O₄/FeVO₄/modified zeolite with a 2:1 ratio) exhibited excellent adsorption and removal properties, effectively eliminating 100 % of the sulfur contaminants. The sulfur removal values for the synthesized samples are also provided in Fig. 17b. As seen in Fig. 17b, sample HS8 exhibits higher photocatalytic efficiency compared to the other samples. Therefore, its ability to desulfurize diesel fuel was further investigated. The results showed that sample HS8 successfully removed 94 % of sulfur within 45 min of irradiation, yielding a highly favorable outcome. Compared to previous studies, this paper presents the first-ever synthesis of a three-component MnFe₂O₄/FeVO₄/modified zeolite photocatalyst. This photocatalyst, synthesized using hydrazine as a controlling agent for hydroxide ion formation, incorporates several key features: zeolite enhances adsorption, iron vanadate provides strong photocatalytic activity, and manganese not only contributes photocatalytic properties but also imparts magnetic characteristics. The magnetic properties of manganese facilitate the separation of the photocatalyst from the suspension, thereby accelerating the recovery of the fuel. Based on the features mentioned, the desulfurization performance of the photocatalyst described in this study has been compared with several recently reported photocatalysts (see Table 7). As shown, the photocatalyst synthesized in this work successfully removed all sulfur from a solution with a higher sulfur concentration (800 ppm) in less time than the other photocatalysts.

**Table 7:** Comparison of the desulfurization result of the photocatalyst synthesized in this research with other previous works.

Type of photocatalyst	Type of sulfur solution	Initial sulfur concentration (mg.L^−1^)	Time of desulfurization (min)	Condition of desulfurization	Desulfurization efficiency (%)	Reference
PMo_11_V/NiO/PAN	BT, TH, and DBT	500	60	visible light	97,98, and 99	[47]
CTAC-HPW	DBT, 4-MDBT, and 4,6-DMDBT	100	30	250 W xenon lamp	99.1, 98 and 80.8	[48]
BiVO_4_–CuO-Clinoptilolite	DBT	500	30	400 W Osram lamp	94.7	[49]
Ni_x_Zn_2-x_V_2_O_7_/WO_4_	BT	500	120	500 W visible light/UV	95	[50]
MnCo_2_O_4_/YVO_4_	TPH	600	150	visible light	100	[51]
MnFe_2_O_4_/FeVO_4_/modified zeolite by CTAB	BT	800	45	400 W Osram lamp	100	This research

## 4 Conclusions

In this study, first MnFe_2_O_4_/FeVO_4_ nanocomposite was synthesized through a facile hydrothermal method using hydrazine and then spread on modified zeolite plates with the help of ultrasonic waves. The products were characterized by XRD, EDS, FTIR, FESEM, TEM, BET, and DRS analyzes and then their photocatalytic performance was evaluated for methylene blue (MB) degradation and benzothiophene desulfurization. Based on the obtained results, it has been observed that both the crystal structure and the chemical composition significantly influence the pollutant removal process. Furthermore, altering the component ratios enhances the efficiency of pollutant removal. Analyzing the results from BET and DRS, it can be concluded that alongside the degradation process, pollutant adsorption also plays a crucial role. The impact of modified zeolite on the adsorption process is particularly noteworthy. For instance, when comparing MnFe_2_O_4_/FeVO_4_ without modified zeolite to MnFe_2_O_4_/FeVO_4_/modified zeolite (2:1), the removal rates for methylene blue (MB) and sulfur increased significantly from 67 % to 68 %–93 % and 100 %, respectively. Consequently, the adsorption kinetics of sample HS8 (MnFe_2_O_4_/FeVO_4_/modified zeolite (2:1)) were investigated, revealing adherence to both the Sips and Khan models. Moreover, the recoverability of sample HS8 (MnFe_2_O_4_/FeVO_4_/modified zeolite (2:1)) was evaluated under various pH conditions, demonstrating satisfactory performance.

## CRediT authorship contribution statement

**Shima H. Khabbaz:** Writing – original draft, Methodology, Formal analysis. **Ahmad Bagheri:** Writing – review & editing, Supervision, Investigation. **Mehdi Mousavi-Kamazani:** Writing – review & editing, Writing – original draft, Supervision, Resources, Methodology.

## Data availability statements

All data generated or analyzed during this study are included in this published article.

## Declaration of competing interest

The authors declare that they have no known competing financial interests or personal relationships that could have appeared to influence the work reported in this paper.
